# Unraveling the host's immune response to infection: Seeing is believing

**DOI:** 10.1002/JLB.4RI1218-503R

**Published:** 2019-02-18

**Authors:** Brittney N.V. Scott, Tina Sarkar, Rachel M. Kratofil, Paul Kubes, Ajitha Thanabalasuriar

**Affiliations:** ^1^ University of Calgary Department of Physiology and Pharmacology University of Calgary Calgary Alberta Canada; ^2^ Calvin Phoebe and Joan Snyder Institute for Chronic Diseases University of Calgary Calgary Alberta Canada; ^3^ Microbial Sciences MedImmune/AstraZeneca LLC Gaithersburg Maryland USA

**Keywords:** intravital microscopy, bacterial infections, innate immunity

## Abstract

It has long been appreciated that understanding the interactions between the host and the pathogens that make us sick is critical for the prevention and treatment of disease. As antibiotics become increasingly ineffective, targeting the host and specific bacterial evasion mechanisms are becoming novel therapeutic approaches. The technology used to understand host‐pathogen interactions has dramatically advanced over the last century. We have moved away from using simple in vitro assays focused on single‐cell events to technologies that allow us to observe complex multicellular interactions in real time in live animals. Specifically, intravital microscopy (IVM) has improved our understanding of infection, from viral to bacterial to parasitic, and how the host immune system responds to these infections. Yet, at the same time it has allowed us to appreciate just how complex these interactions are and that current experimental models still have a number of limitations. In this review, we will discuss the advances in vivo IVM has brought to the study of host‐pathogen interactions, focusing primarily on bacterial infections and innate immunity.

## Graphical abstract

Reviews the advances in vivo intravital microscopy has brought to the study of host‐pathogen interactions, focusing primarily on bacterial infections and innate immunity.

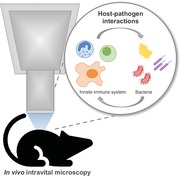

**Abbreviations**
CRIg
: complement receptor of the immunoglobulin superfamily
EPEC: enteropathogenic *Escherichia coli*
GI: gastrointestinal
IELs: intraepithelial lymphocytes
iNKT: invariant natural killer T
IVM: intravital microscopy
NETs: neutrophil extracellular traps
PAD4: peptidylarginine deiminase 4
RFP: red fluorescent protein
TRPV1: transient receptor potential vanilloid 1
UPEC: uropathogenic *Escherichia coli*
UTI: urinary tract infection

## 1 INTRODUCTION

**Figure 1:**
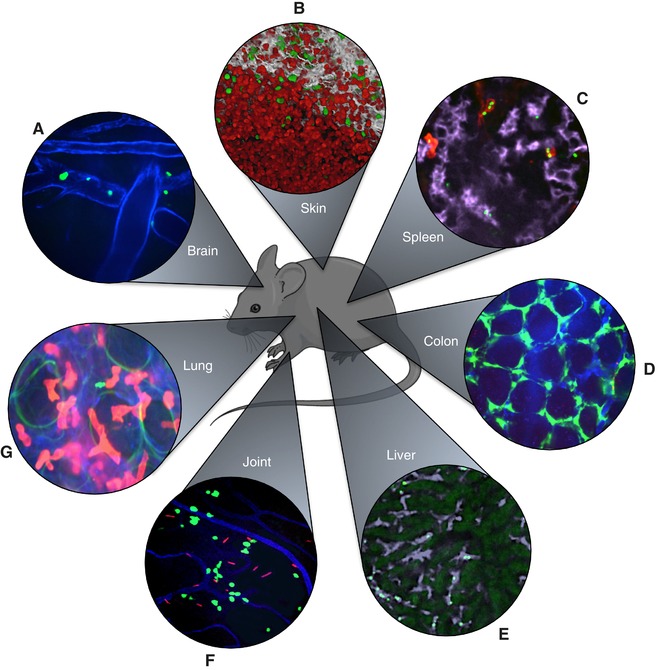
**Visualizing innate immune cell responses to bacterial infections in different organs using intravital microscopy**. (**A**) Brain SDC‐IVM image showing neutrophils (green, LysM‐eGFP) rolling in cerebral vessels (blue, anti‐CD31) 4 h after intracerebroventricular injection of LPS. (**B**) Skin MP‐IVM image showing neutrophils (red, tdTomato) localized to the center of a *Staphylococcus aureus* skin infection, with monocytes/macrophages (green, CX_3_CR1‐GFP) distributed around the perimeter of the infection. Collagen is visualized in white using second harmonic generation. (**C**) Spleen SDC‐IVM image showing splenic red pulp macrophages (magenta, anti‐F4/80) and neutrophils (red, anti‐Ly6G) capturing blood‐borne *Streptococcus pneumoniae* (green, GFP bacteria). (**D**) Colon SDC‐IVM image of the colonic lamina propria after *Salmonella typhimurium* (red, mCherry bacteria) infection, with macrophages (green, CX_3_CR1‐GFP) localized in proximity to the microvasculature (blue, anti‐CD31) surrounding the intestinal crypts. (**E**) Liver SDC‐IVM image showing Kupffer cells (magenta, anti‐F4/80) in the liver sinusoids (dark areas) catching blood‐borne *Staphylococcus aureus* (bright green, GFP bacteria). Hepatocytes are visualized as dim green autofluorescence. (**F**) Knee joint SDC‐IVM image showing iNKT cells (green, CXCR6‐GFP) interacting with *Borrelia burgdorferi* (red, tdTomato bacteria) in the joint 3 days after systemic infection. Vasculature is shown in blue (anti‐CD31). (**G**) Lung SDC‐IVM image showing neutrophils (red, anti‐Ly6G) in the pulmonary vasculature (blue, anti‐CD31) interacting with *Streptococcus pneumoniae* (bright green, GFP bacteria) after systemic infection. Alveoli are visualized as green autofluorescent rings. eGFP, enhanced green fluorescent protein; iNKT, invariant natural killer T; IVM, intravital microscopy; LPS, lipopolysaccharide; LysM, lysozyme M; MP, multiphoton; SDC, spinning‐disk confocal

Since the first study using intravital microscopy (IVM) was published in the 1800s, the technology has progressed significantly.^1^, ^2^ We have moved away from simple light microscopy of the frog tongue to sophisticated multiphoton laser systems, allowing us to image most organs in mammals. These advances have enabled scientists to look inside the body of living organisms and examine organs such as the brain, colon, spleen, liver, skin, joint, and even the lung (Fig. 1). Murine models are the most commonly used and well established for IVM; however, many species are amenable to this approach.^3^ In this review, we will discuss the recent discoveries IVM has brought forth to the world of host‐pathogen interactions, specifically focusing on the innate immune system's response to bacterial infection. IVM does not only provide us with beautiful images and videos, but more importantly, it allows us to understand dynamic cell‐cell interactions and spatiotemporal events key to the clearance of infections. Just in the past decade, IVM has unraveled many important and novel immune processes. These include, for instance, the swarming behavior of neutrophils,^4^, ^5^, ^6^ pathogen dissemination by neutrophils and macrophages,^7^, ^8^ and previously unrecognized bacterial reservoirs following infection.^9^

Multiphoton and confocal microscopes are both widely used for IVM and each has certain advantages over the other. Multiphoton instruments excite fluorophores in the specimen using two (or more) photons delivered by high‐intensity light.^10^ The main advantages are: deeper tissue penetration, minimal out‐of‐focus photodamage, and the ability to excite endogenous molecules (e.g., collagen).^1^, ^10^ Although confocal microscopes cannot penetrate as deep, certain instruments such as spinning‐disk confocal systems can capture rapid events, effectively in real time.^1^, ^11^ A limitation of IVM is that you only see what you label. With the very recent advent of the white light confocal laser and spectrally tunable multiphoton system, comes the flexibility to tune across a full spectral range. This will allow for the use of more fluorophores and thus, the ability to visualize more cell types during IVM.

The immune cells best studied using IVM are neutrophils, monocytes/macrophages, dendritic cells, T cells, and invariant natural killer T (iNKT) cells, as lineage‐specific antibodies and reporter mice have been developed to label these cells effectively in vivo.^12^ Fluorescently labeled antibodies specific for different cell‐surface markers are valuable tools for effectively tagging a range of cell types, including immune cells, endothelial cells, and epithelial cells. However, when using antibodies, the route of delivery needs to be considered to ensure that the cell of interest will actually be labeled. For instance, intravenous antibodies will not effectively label cells in the brain under normal conditions due to exclusion by the blood‐brain barrier. Moreover, many antibodies do not work well for in vivo imaging, even if they are effective for other techniques such as flow cytometry, and the fluorophores used to label the antibodies are typically more susceptible to photobleaching than expressed reporter proteins. Transgenic mice with a fluorescent reporter protein, such as GFP or red fluorescent protein (RFP), inserted into a gene of interest allows for the visualization of specific cell types for prolonged lengths of time in different tissues and conditions. A limitation, however, is that many reporter strains report on more than one cell type. Thus, another method used by researchers to overcome some of these limitations and to study the long‐term fate of specific cells is adoptive transfer. Here, a cell type of interest is isolated from a fluorescent animal (e.g., by fluorescence activated cell sorting) and transferred into a nonfluorescent animal. This method allows the tracking of fluorescent cells throughout the body, which can provide valuable information about where a particular cell homes to, during infection for instance. Yet, adoptive transfer also comes with a set of limitations as ex vivo sorting can often have pleiotropic effects on the cells that are harvested. Fluorescent strains of many different types of bacteria, expressing proteins like GFP or RFP, are widely available and used for IVM. Membrane‐permeable fluorescent dyes, such as SYTO9, can also be used to label viable bacteria for imaging; however, these labels are diluted as the bacteria replicate. Novel imaging techniques to track bacteria that replicate, die, or become persisters are also slowly becoming available^13^, ^14^ and, when applied in vivo, will report on essential microbial biology. Using a combination of the techniques described above, researchers have used IVM to significantly advance our understanding of host‐pathogen interactions in many different organs to many different types of infections.

Yet, IVM is no longer just a tool used merely to understand fundamental immunology. It has now become an important technique used in the development of novel drug therapies.^15^ For example, after seeing that *Staphylococcus aureus* hides inside liver‐resident Kupffer cells making conventional intravenously administered vancomycin ineffective, researchers used IVM to study the effectiveness of novel drug delivery methods (i.e., liposomes loaded with vancomycin) to eradicate the shielded bacteria.^9^ As we learn more about which cells are involved in the clearance of specific pathogens and what triggers pathogens to adopt chronic infectious modes, the more IVM will be used as a drug discovery and validation tool. In this review we describe the findings that IVM has brought forth to the fields of immunology and bacteriology to better understand host‐pathogen interactions. We focus on key discoveries reported in recent studies, and have divided the review based on the organ imaged and the infection model used (i.e., systemic infections versus localized infections).

## 2 SYSTEMIC INFECTIONS

### 2.1 Systemic infections: Imaging the lung

Pneumonia is one of the leading causes of hospital admissions in North America.^16^ Thus, the ability to image the lungs of live mice was an important turning point in the fields of immunology and infectious disease. Although there are publications on lung imaging that go back 40 years, a study published in 2011^17^ made lung IVM much more user‐friendly by developing a simple window with gentle suction that is placed on the exterior of the lung after opening the thoracic cavity. By stabilizing the lung of live, breathing animals, this technique could be used to visualize dynamic cell behaviors in vivo.^17^ This study sparked a new interest in studying pulmonary immune responses in various conditions using IVM. The biggest challenge, apart from accessing and stabilizing the lung, is that the airways are difficult to image due to the visual barrier of the air‐liquid interface. Thus, most researchers have focused on imaging the behavior and function of innate immune cells within the liquid phase (i.e., the vasculature), under basal conditions and after a systemic or local lung infection.

At steady state, lung‐resident neutrophils are found crawling inside the capillaries and are in constant contact with the lung endothelium. A recent study by Yipp et al.^18^ sought to understand the dynamic relationship between these resident immune cells and the endothelium. Using IVM, neutrophils were observed interacting with lung capillaries (but not larger vessels) by tethering, crawling, or remaining adherent to the capillary walls.^18^ After the systemic administration of lipopolysaccharide, the majority of neutrophils in the lung rapidly took on an activated, crawling phenotype. Molecular investigation showed that this rapid neutrophil activation and crawling phenotype was dependent on increased surface expression of CD11b mediated by TLR4‐MyD88 and abl‐kinase pathways.^18^ In a more physiologic model of infection with *Escherichia coli*, Yipp et al. showed that these pathways, where effector responses were turned on in minutes, play a crucial role in the rapid capture of *Escherichia coli* during bloodstream infections.^18^

Thanabalasuriar et al.^19^ used IVM to study another pathogen, *Pseudomonas aeruginosa*. In this study, *Pseudomonas aeruginosa* was observed adhering to the lung vasculature during systemic infection and, remarkably, resident patrolling neutrophils were unable to recognize and clear the bacteria. In this study bacterial mechanisms behind this cloaking pheontype were uncovered. It was found that a virulence factor, specifically the exopolysaccharide Psl, allowed *Pseudomonas aeruginosa* to cloak itself from the neutrophils. Targeting this virulence factor with a therapeutic antibody unveiled the bacteria to the neutrophils and allowed phagocytosis.^19^ Interestingly, *Pseudomonas aeruginosa* utilized another virulence factor, the type III secretion system, to secrete effector molecules that hindered intracellular killing by the neutrophils. Thus, a bispecific antibody targeting both virulence factors enabled the effective clearance of this pathogen.^19^

Blood‐borne infections and sepsis are known to initiate a complex inflammatory response in the lung, and IVM has helped us better understand these processes. Schmidt et al.^20^ sought to investigate the mechanisms involved during sepsis‐induced acute lung injury. The authors found that endotoxemia and experimental sepsis rapidly induced the degradation of the pulmonary microvascular glycocalyx and loss of heparan sulfate through TNF‐α‐dependent mechanisms. This increased the availability of endothelial surface adhesion molecules and, thus, contributed to neutrophil adhesion and subsequent lung injury.^20^ Indeed, inhibiting the degradation of heparan sulfate was demonstrated to significantly attenuate lung injury and improve survival. Although the greatest protection was observed when animals were prophylactically treated with heparin or were deficient for heparanase (*Hpse^−/−^*), a protective effect was still observed when treatment was delayed to 24 h after sepsis induction.^20^

It is clear that a balance is needed, as excessive inflammation can cause tissue damage whereas blocking the ability of the innate immune system to clear invasive pathogens can lead to overwhelming infection. For example, Lee et al.^21^ found that blocking leukotriene B4 in a lethal *Candida albicans* sepsis model allowed neutrophils to clear the fungi and ameliorate infection‐induced capillaritis in the lung. Clearly, research using IVM should help to determine which targets are effective for which pathogens at which time points, and improve current therapeutic strategies for patients with pulmonary infections and sepsis‐induced lung injury.

Additionally, the more that immunologic responses are studied in different tissues, the more it is appreciated that different vascular beds have unique endothelial properties. For instance, although heparan sulfate was shown to be important for leukocyte‐endothelial cell interactions in the pulmonary microvasculature,^20^ a similar role was not found in the peritoneal microvasculature.^22^ Thus, it highlights that the conclusions drawn from one organ, such as early studies using tissues like the cremaster muscle to study leukocyte recruitment, cannot necessarily be applied to other organs, and that organ‐specific IVM will continue to be an incredibly important technique.

### 2.2 Systemic infections: Imaging the spleen

Although humans can live without a spleen, splenectomy increases the risk for sepsis and meningitis caused by encapsulated bacteria, such as *Streptococcus pneumoniae*, *Neisseria meningitidis*, and *Haemophilus influenzae* type B.^23^, ^24^  Despite being an important immune organ, few studies to date have used IVM to image the spleen. One of the first studies, published in 2008 by Aoshi et al.,^25^ used IVM to examine the splenic immune response after a systemic *Listeria monocytogenes* infection. This landmark study demonstrated that splenic dendritic cells help to initiate rapid CD8^+^ T cell responses to *Listeria monocytogenes* by transporting the bacteria from the marginal zone of the spleen to the T cell areas of the white pulp.^25^

In a more recent study, Deniset et al.^26^ used IVM to investigate the coordinated response of splenic neutrophils, macrophages, and B cells to fight *Streptococcus pneumoniae*. They found that, during a systemic *Streptococcus pneumoniae* infection, most of the bacteria bypass the marginal zone macrophages and are caught by the red pulp macrophages. Mature Ly6G‐high neutrophils residing in the red pulp (that were observed to scan the tissue under basal conditions) were seen plucking the bacteria off the surface of the red pulp macrophages.^26^ A dramatic increase in neutrophils was also observed in the marginal zone by 24 h after infection, and interestingly, IVM showed that these neutrophils were grabbed right out of the bloodstream by the marginal zone macrophages. Deniset et al. demonstrated that these retained neutrophils interacted with marginal zone B cells to promote thymus‐independent antibody production, which further enhanced the eradication of *Streptococcus pneumoniae*.^26^

The spleen plays an important role in the filtration of blood‐borne bacteria. However, it can also serve as a permissive reservoir for pathogens such as *Streptococcus pneumoniae*.^27^ Acting as a site for both innate and adaptive immune processes to take place, multiple immune cell populations including neutrophils, monocytes, red pulp macrophages, marginal zone macrophages, dendritic cells, B cells, T cells, and NKT cells reside within the splenic red pulp, marginal zone, and white pulp.^23^, ^26^ How these numerous cell types behave and interact in the spleen over the course of infection with different pathogens is still unclear and, thus, IVM will provide important insight into these mechanisms.

### 2.3 Systemic infections: Imaging the liver

Whether examining acute injury, chronic disease, or infection, the liver has been one of the most successfully imaged internal organs. Many studies have investigated Kupffer cells, the resident liver macrophages, and have found that these cells play a vital role in the capture and clearance of many different types of bacteria from the blood, including *Staphylococcus aureus*, *Escherichia coli*, *Borrelia burgdorferi*, and *Listeria monocytogenes*, but not encapsulated bacteria such as *Streptococcus pneumoniae*.^28^, ^29^, ^30^, ^31^, ^32^, ^33^, ^34^ The importance of several receptors, including scavenger receptors and the complement receptor of the immunoglobulin superfamily (CRIg), as well as platelet binding in fast versus slow track catching pathways has been described using IVM.^28^, ^33^ However, Kupffer cells cannot always effectively kill the bacteria they catch and can be overcome by pathogens such as *Listeria monocytogenes* 35 and methicillin‐resistant *Staphylococcus aureus*.^9^ Two recent reviews summarize the literature focused on immune responses in the liver and specifically discuss the use of IVM to understand the role of the liver in the clearance of blood‐borne infections.^1^, ^36^ Thus, in the current review we will just highlight a number of recent findings.

Despite being tiny, platelets can stimulate a robust immune response and have been shown to play an important role during infection.^37^ Platelets can help trap and bundle circulating bacteria to promote infection clearance^38^, ^39^; albeit, this can similarly cause significant microvascular dysfunction and tissue injury. In a recent study, Surewaard et al.^40^ used IVM to investigate the mechanisms and treatment of microvascular dysfunction and thrombosis associated with systemic *Staphylococcus aureus* infections. The group found that platelets rapidly formed aggregates after exposure to alpha‐toxin secreted by *Staphylococcus aureus*. This caused damaging thrombosis throughout the liver and subsequent organ dysfunction.^40^ Neutralizing alpha‐toxin with a therapeutic antibody effectively prevented platelet aggregation and liver damage, without affecting initial bacterial capture, and thus offered a new approach that may help combat staphylococcal‐induced microvascular coagulation and organ dysfunction.^40^

Another study used liver IVM to examine sex‐biased differences in the capture of blood‐borne bacteria by Kupffer cells.^30^ Interestingly, Zeng et al.^30^ found that while male mice relied on complement opsonisation to capture systemic enteropathogenic *Escherichia coli* (EPEC), females were privy to faster capture due to preexisting natural antibodies against EPEC. The production of these antibodies was found to be dependent on a sex hormone‐driven pathway during puberty, which did not require overt immunization or microbial colonization. Moreover, the maternal transfer of the antibodies through milk conferred protection to offspring.^30^ This work highlights an evolutionary strategy developed by the female host to protect herself and her offspring from this threatening pathogen.

Given the number of functions the liver is tasked with, this organ offers the potential to study many aspects of health and disease ranging from homeostasis to infection, injury, autoimmunity, chronic disease, cancer, healing, and regeneration. Using IVM, researchers can visualize the various cell types of the liver and those in the circulation, including hepatocytes, sinusoidal endothelial cells, Kupffer cells, dendritic cells, neutrophils, lymphocytes, and platelets, and gain insight into their role and fate over time in numerous experimental conditions.

### 2.4 Systemic infections: Imaging the brain

The brain can be directly or indirectly affected by infection. Although the blood‐brain barrier and blood‐cerebrospinal fluid barrier offer protection from the circulation, certain pathogens can penetrate these cellular barriers and enter into the brain.^41^ Pathogens may also use other routes, such as translocation from the nasal mucosa, or gain access to the brain following a traumatic injury.^41^ A number of studies have used IVM to better understand the pathogenesis of and immune responses to several different types of viral,^42^ parasitic,^43^, ^44^, ^45^ and fungal^46^ infections of the brain and meninges. For instance, IVM of the brain during *Toxoplasma gondii* infection revealed that this parasite gains access to the central nervous system by invading, replicating in, and lysing endothelial cells,^45^ and it is possible that other pathogens (e.g., *Listeria monocytogenes*) also use this pathway.

Once inside the brain parenchyma, resident microglia rapidly respond to the invading pathogen and can recruit additional leukocytes, such as neutrophils, monocytes, and CD8^+^ T cells, from the circulation.^41^ IVM of the brain has helped us better understand the dynamics of leukocyte recruitment into the central nervous system during injury and infection, yet the number of studies focused on imaging bacterial infections in the brain is limited and many questions remain. IVM of the brain is complicated by the fact that the brain is encased in a rigid skull. Thinning or removing a section of the skull enables the visualization of the meninges and parenchyma, yet how deep into the parenchyma one can see is limited by current microscope capabilities. Now, with more sophisticated multiphoton systems available, it will be possible to image deeper into the cortex to reveal new host‐pathogen interactions that to date have not been observed. Moreover, the blood‐brain barrier hampers the ability to use intravenously administered fluorescent antibodies, thus the development of transgenic animals expressing fluorescent proteins (e.g., CX_3_CR1^+/GFP^ mouse to study microglia) has been seminal for IVM studies of the central nervous system. For a long time, the brain was described as an immune‐privileged organ, yet IVM has allowed us to directly visualize the dynamic immune environment during health and disease, challenging this simplified concept. A number of bacteria, such as *Streptococcus pneumoniae*, *Neisseria meningitidis*, and *Listeria monocytogenes*, continue to be important causes of central nervous system infections,^41^ yet further work is needed to fully characterize the complex immune‐microbe interactions that occur once these pathogens infect this important organ.

Notably, it has become apparent that inflammation triggered by systemic infections can negatively impact the brain and two recent studies used IVM to examine these changes after bacterial sepsis.^47^, ^48^ Plotkowski et al.^48^ used a *Pseudomonas aeruginosa* pneumonia model of sepsis and observed significant leukocyte rolling and adhesion to the cerebral vessels accompanied by impaired capillary perfusion in the brain. Moreover, they showed that these effects were primarily mediated by the release of the cytotoxin ExoU by *Pseudomonas aeruginosa*, which initiated an inflammatory response in the cerebral vasculature by activating the platelet‐activating factor receptor pathway.^48^ Another study, by Andonegui et al.,^47^ imaged the brains of mice following *Streptococcus pneumoniae* pneumonia. The authors observed an increased recruitment of neutrophils and CCR2^+^ inflammatory monocytes into the brain, as well as the subtle activation of microglia. These events transpired in the absence of any bacteria detected in the brain.^47^ Interestingly, it was found that inhibiting the recruitment of monocytes, but not neutrophils, significantly reduced signs of neuroinflammation and cognitive impairment after infection.^47^ This is in line with numerous recent non‐IVM publications implicating monocytes in brain inflammation.^49^, ^50^, ^51^

### 2.5 Systemic infections: Imaging the joints

It is well known that certain types of bacteria home to the joints, such as Lyme disease‐causing *Borrelia*, yet our understanding of the immune responses to these infections in the joint is still limited. Two studies have used IVM to image the joints of mice after infection with *Borrelia burgdorferi* and have provided novel insights into the key cellular players and dynamic host‐pathogen interactions. Lee et al.^52^ imaged the knee joint of mice after systemic *Borrelia burgdorferi* infection. Interestingly, they found that extravascular joint‐resident iNKT cells played an important immune surveillance role in this tissue and were critical for defending the joint against these bacteria. iNKT cells are important responders to various types of infections. These cells are potent producers of cytokines, have cytotoxic activity, and can be activated directly by recognizing bacterial glycolipids through their invariant T cell receptor or indirectly by cytokines and TLRs.^53^, ^54^ In the study by Lee et al., iNKT cells were observed crawling close to the joint blood vessels and directly interacting and killing invading spirochetes, which was found to be granzyme dependent.^52^ Notably, iNKT cell‐deficient animals have a significantly higher burden of bacteria and inflammation in the joint after infection.^52^, ^55^ Humans are particularly susceptible to Lyme arthritis, thus Kumar et al.^56^ used IVM to further study the vascular transmigration of *Borrelia burgdorferi* into the joints using iNKT cell‐deficient mice. They identified P66, a bacterial integrin adhesin and porin, to be required for vascular transmigration into the joint tissue by this spirochete.^56^

As discussed here, certain bacterial pathogens like *Borrelia* can home to the joints during a systemic infection; however, another common cause of joint infections is prosthetic surgery. Over a million prosthetic surgeries are performed each year and the incidence of infection ranges from 1% to 4%, with more than 50% of these infections caused by *Staphylococcus aureus* and coagulase‐negative staphylococci.^57^ Thus, further investigations into the role of the immune system in clearing bacteria from the joint are warranted. Moreover, whether iNKT cells play a role in preventing dissemination from the joint into the vasculature in these types of infections is also worth examining.

Joint inflammation due to injury and chronic disease also causes significant morbidity and disability in humans. IVM has been used to better understand the dynamics of neutrophil recruitment into the joints during immune complex‐induced arthritis.^58^ However, many questions remain and IVM will continue to be a powerful tool to answer specific questions regarding: (i) the spatiotemporal recruitment dynamics of immune cells into the joints; (ii) the role of joint‐resident immune cells, such as iNKT cells, in various conditions; and (iii) how the immune system can be modulated to reduce inflammatory injury and improve tissue healing.

## 3 LOCALIZED INFECTIONS

### 3.1 Localized infections: Imaging the lung

As discussed above, IVM has been used by researchers to better understand the pulmonary immune responses to systemic infections. On the other hand, there are a number of studies that have used IVM to study local lung infections (i.e., infection models where the bacteria are introduced into the airways). Initially, studies using explanted whole lungs and micromanipulation to place bacteria directly into the alveoli were used and have provided important insights into our understanding of bacterial pathogenesis in the lung's airspaces.^59^, ^60^, ^61^ However, removing the lungs from their native environment and inflating them with agarose does not recapitulate in vivo physiology. Therefore, advances in the techniques that have enabled researchers to image the lungs of living, breathing animals have been critical in this field.

Neutrophils can utilize several methods to help clear infections. One of these mechanisms includes the release of neutrophil extracellular traps (NETs), web‐like structures of decondensed DNA and proteins (e.g., histones, neutrophil elastase, myeloperoxidase, and proteases) that can trap and kill pathogens.^62^ NET formation requires the peptidylarginine deiminase 4 (PAD4) enzyme, which plays a role in histone citrullination and chromatin decondensation, and mice deficient for this enzyme cannot make NETs.^63^, ^64^ However, it is challenging to study NETs in vivo, as it is difficult to discriminate NETs from other cell‐free or bacterial‐derived DNA at sites of infection. Moreover, rare events can make it difficult to capture neutrophils undergoing NETosis. Although NETs have been shown to play an important role during infection, they can also have detrimental effects on the surrounding tissue. A recent study by Lefrancais et al.^65^ showed that a balance is indeed needed during severe bacterial pneumonia. Using two‐photon IVM, this group was able to visualize, for the first time, the formation of NETs in vivo in the lung after infection. In their model of infection‐induced lung injury (instillation of a high dose of methicillin‐resistant *Staphylococcus aureus*), NETs were formed in the airspaces and also in the microvasculature, leading to poor survival of the animals due to overt lung injury.^65^ On the other hand, when mice were unable to produce NETs in response to the infection (PAD4^−/−^), lung injury was indeed reduced; however, bacterial clearance was significantly impaired. This similarly led to poor survival of the animals. Interestingly, when a balance was reached in the heterozygous PAD4^+/−^ mice, which had intermediate NET production, survival of the animals was significantly improved. Intratracheal DNase treatment after infection was also effective at reducing lung injury and improving survival.^65^ This study highlights the importance of IVM as a technique to help better understand the pathophysiology of infections in order to develop effective therapeutics.

A novel area of immunology research is the neural regulation of immunity. It has been established that the lung is innervated, and crosstalk between immune cells in the lung and nociceptors help drive allergic responses and bronchoconstriction during asthma.^66^, ^67^ Recently, Baral et al.^68^ showed that TRPV1^+^ nociceptor neurons crosstalk with neutrophils in the respiratory tract, which has a detrimental effect on survival and outcome during lethal *Staphylococcus aureus* pneumonia. The authors used IVM to study neutrophil dynamics in this model and found that blocking TRPV1^+^ neurons with a pharmacologic inhibitor allowed neutrophils to crawl longer distances, enhancing their function to eradicate *Staphylococcus aureus*.^68^ The findings of this study highlight the potential for targeting the immune system via the nervous system to improve outcomes after infection.

IVM is a powerful tool to study the spatial distribution of coordinated immune responses involving multiple immune cell types. A study published in 2014 imaged the lungs during anthrax infection to evaluate interactions between alveolar macrophages and dendritic cells.^69^ This group used a technique to image nonstabilized lungs through the thoracic cavity (correcting movement *a posteriori*) and observed that the administration of *Bacillus anthracis* spores induced long‐lasting interactions between alveolar macrophages and dendritic cells. A limitation of this method, however, is that without stabilization of the lung (e.g., using a thoracic window and vacuum^17^), dynamic cell‐cell interactions may be missed and 3D imaging is not possible. Thus, it can be difficult to determine the exact location of cells and assess whether they are truly interacting, rather than existing in different *z*‐planes even if *xy*‐planes appear to overlap.

Thanabalasuriar et al.^70^ used the stabilized lung IVM technique to understand how innate immune cells, including iNKT cells, communicate within the different pulmonary compartments to resolve a *Streptococcus pneumoniae* infection. iNKT cells, which predominantly reside in the lung vasculature, were found to migrate out of the vasculature and into the interstitial space. This movement was found to be dependent on neutrophils, which helped guide iNKT cells out of the vasculature by releasing the chemokine CCL17.^70^ In the interstitial space, monocyte‐derived dendritic cells presented antigens to the newly extravasated iNKT cells, leading to their activation and retention in this location, whereas the neutrophils continued into the airways. Blocking the movement or activation of iNKT cells increased the susceptibility to *Streptococcus pneumoniae* infection.^70^ Imaging provided the necessary evidence to be able to understand why iNKT cell‐deficient mice are so susceptible to this pathogen.

The lungs are in constant contact with the external environment and, thus, have an important homeostatic immunologic function. In the lung, immune cells can reside within the airspaces (e.g., alveolar macrophages), pulmonary vasculature (e.g., neutrophils), or between these two compartments in the interstitium (e.g., dendritic cells).^71^ The movement of cells between compartments is necessary for the clearance of certain pathogens, yet has also been shown to promote dissemination. For instance, a non‐IVM study recently reported that the movement of infected alveolar macrophages into the interstitium may drive the dissemination of *Mycobacterium tuberculosis*.^72^ Although an interesting hypothesis, IVM will be necessary to track the migration of these cells to demonstrate categorically that it is indeed the alveolar macrophages that migrate into the interstitium. IVM imaging of live events such as this will help to improve our understanding of the dynamic host‐pathogen interactions that take place during infection with different types of intracellular and extracellular bacteria.

### 3.2 Localized infections: Imaging the gastrointestinal tract

The gastrointestinal (GI) tract is a hotbed for host‐bacterial interactions, and the status of its microbiome is known to influence multiple aspects of health and disease, including infection, obesity, autoimmune disease, and cancer.^73^ Thus, research groups that have developed IVM techniques to image the GI tract pave the way for a deeper understanding of the critical interplay that occurs between the immune system and the diverse bacterial species residing in these organs. However, this has not been without a number of challenges. Typically, researchers are interested in imaging the luminal side of the GI organs and therefore need to invasively manipulate the tissue, which is technically difficult and can have unwanted effects. Moreover, constant movement of the tissue due to peristalsis makes it difficult to obtain stable videos. Thus, advances in software analysis tools enabling correction of this movement have been important to allow for the visualization of dynamic events, such as cell‐cell interactions.^74^

Stomach ulcers are a common cause of gastric tissue damage and are often associated with *Helicobacter pylori* infection.^75^ The pathogenesis of this bacterium has been studied using IVM by Aihara et al.,^76^ who were interested in understanding how *Helicobacter pylori* affects wound healing in the stomach. To model a localized ulcer‐type injury, the authors injured the gastric surface using a two‐photon laser and imaged the repair process over time. It was observed that *Helicobacter pylori* preferentially colonized the ulcerated areas, by rapidly crawling toward the damaged tissue, and significantly impaired wound healing.^76^

The intestine houses many different resident immune cell populations, including macrophages, dendritic cells, mast cells, eosinophils, T cells, B cells, and various innate‐like lymphocytes, which play important homeostatic and protective roles.^77^ Intraepithelial lymphocytes (IELs) are specialized immune cells that reside close to the epithelial cell layer.^78^ A recent study using IVM looked into the behavior of these IELs in response to the gut microbiota and during infection.^79^ In the steady state, IELs were found to be actively motile, surveying the intestinal epithelial cells. In germ‐free mice, however, the difference was striking. IELs were less motile and had lost directional movement, which suggests that IELs depend on the presence of commensals to survey the intestine.^79^ During infection with *Salmonella typhimurium* or *Toxoplasma gondii*, IELs were seen not only scanning the intestinal wall, but taking on another behavior pattern that the authors termed “flossing.” This flossing movement allowed IELs to squeeze in between the intestinal wall cells at locations that were clustered with pathogens. IEL surveillance and flossing behaviors were dependent on epithelial cell MyD88 signaling.^79^

Resident macrophages and dendritic cells sample the intestinal luminal content by extending dendrites between epithelial cells and can respond quickly during infection.^80^, ^81^, ^82^ Following challenge with *Salmonella typhimurium*, CX_3_CR1^−^ CD103^+^ dendritic cells were observed to concentrate in the epithelium and efficiently phagocytose bacteria using intraepithelial dendrites to pull bacteria from the lumen.^82^ This sampling process by intestinal dendritic cells was important for driving subsequent adaptive immune responses to *Salmonella typhimurium* in mesenteric lymph nodes.^82^ On the other hand, CX_3_CR1^+^ cells were found to rapidly migrate into the intestinal lumen at locations close to *Salmonella* clusters and helped control the initial infection.^83^

IVM imaging of the GI tract has significantly improved over the last several years.^84^ The refinement of surgical techniques and tissue preparations, as well as microscope capabilities, has allowed researchers to peer into these dynamic environments that house constant host‐microbe interactions.^84^ Although the aforementioned studies highlight the power of IVM, there still remain large gaps in the literature that need to be addressed going forward. For example, Aihara et al.^76^ were limited to imaging the serosal side of the stomach; however, ulcers and *Helicobacter pylori* infections typically affect the mucosal surface. Further, IVM studies imaging the colon and rectum after infection are lacking. Addressing these gaps will not only progress the study of cell interactions in the GI tract, but also aid in finding therapeutic targets for GI tract‐related diseases and infections. Moreover, the mounting interest in the microbiome is driving the establishment of state‐of‐the‐art germ‐free facilities, which will allow scientists to answer very specific questions about the critical relationship between the immune system and different microbes using IVM.

### 3.3 Localized infections: Imaging the skin and skin‐draining lymph nodes

The skin is the body's largest organ. It is an active and protective barrier against the external environment. The skin has a dynamic and complex immune network that helps maintain its barrier function and protect against infection when needed.^85^ IVM has improved our understanding of the cutaneous immune system, as well as the immune responses that take place in the skin‐draining lymph nodes, during different types of infections. The two most commonly used models to image skin immune responses include the ear skin model and the exteriorized dorsal skin flap model.

A number of IVM studies have used *Staphylococcus aureus* infection models to study immune responses in the skin. *Staphylococcus aureus* is a major cause of severe skin and soft tissue infections in humans.^86^ As a highly invasive pathogen, efficient control and clearance of *Staphylococcus aureus* in the skin is necessary to prevent dissemination and the development of sepsis. During a local skin infection, neutrophils rapidly extravasate from dermal blood vessels and crawl toward the infection focus, a process that is dependent on G‐protein coupled receptors.^87^ In 2014, Abtin et al.^88^ identified a critical, and previously unrecognized, role for perivascular macrophages in the recruitment of neutrophils using IVM. It was found that perivascular macrophages, which closely associate with dermal venules, are major producers of neutrophil chemoattractants. Moreover, the authors showed that α‐hemolysin, an important toxin produced by *Staphylococcus aureus*, lysed these perivascular macrophages, which in turn reduced neutrophil recruitment to the skin and helped *Staphylococcus aureus* evade clearance by the immune system.^88^ In another study, Harding et al.^89^ found that neutrophils began crawling in the capillaries of the skin when *Staphylococcus aureus*‐coated beads were injected into the subcutaneous tissue. The beneficial reason for this behavior remains unclear as the neutrophils crawling inside capillaries impaired capillary perfusion and increased parenchymal cell death. Blocking the β_2_ and α_4_ integrins reduced the number of neutrophils crawling within the capillaries, improved capillary perfusion, reduced cell death, and decreased lesion size after infection, associating this behavior with pathology.^89^

A different study, which focused on visualizing the production of NETs in the skin during *Staphylococcus aureus* infection, used IVM to characterize a novel mechanism.^90^ In response to *Staphylococcus aureus*, neutrophils recruited to the skin were able to rapidly produce an abundant amount of NETs.^90^ The key finding of this in vivo study, however, was that NETs were released by live, crawling neutrophils. This type of “vital NETosis” (distinct from lytic NETosis) allowed neutrophils to multitask during a *Staphylococcus aureus* skin infection, which prevented bacterial dissemination and bacteremia.^90^

IVM has also been used to better understand the initial infection and immune response to *Yersinia pestis*, the bacterial pathogen responsible for causing plague that is introduced into the skin by the bite of an infected flea.^91^, ^92^ Shannon et al.^91^, ^92^ used two models of *Yersinia pestis* infection where the bacteria were either administered by intradermal injection or naturally by flea bite. When *Yersinia pestis* (1000 CFU) was injected into the skin, a rapid and robust neutrophil response was observed using IVM.^92^ IVM was important for visualizing this very localized neutrophil response, which could not be detected by flow cytometry.^92^ During natural infection by flea bite, the immune response to *Yersinia pestis* appeared to differ depending on the amount of bacteria transmitted, where neutrophils dominated when high numbers of bacteria were transmitted and macrophages dominated when low numbers of bacteria were transmitted.^91^

Several studies have used IVM to study the role of the innate immune system in the draining lymph nodes of the skin during infection. After the introduction of heat‐killed^93^ or live^94^, ^95^ *Staphylococcus aureus* into the skin, neutrophils are rapidly recruited to the draining lymph nodes. Hampton et al.^93^ showed that neutrophils migrated from the inflamed skin into the lymph node via the lymphatic vessels where they helped modulate lymphocyte proliferation. Bogoslowski et al.^94^ imaged the draining lymph nodes of mice following *Staphylococcus aureus* skin infection and identified a robust complement‐dependent neutrophil recruitment. However, in this study, neutrophils were found to enter the lymph node primarily from the blood via high endothelial venules and helped to intercept *Staphylococcus aureus* and prevent dissemination.^94^ The differing results may be an issue of different doses, volumes, or strains of *Staphylococcus aureus* used, but may also reflect the caveats of imaging. You only see what you look at and, as such, if one only examines a single compartment, it can generate an incomplete picture. Although neutrophil infiltration into the lymph node is thought to be a protective mechanism used by the host, it has been shown to limit local humoral responses through direct neutrophil‐B cell interactions that suppress the production of IgM.^95^ Moreover, the infiltration of immune cells can cause tissue injury and disrupt the organization of the lymph node, limiting beneficial interactions between subcapsular sinus macrophages and B cells.^96^ Remarkably, lymphatic impairment following a localized methicillin‐resistant *Staphylococcus aureus* skin infection can be sustained long after the infection has been cleared and the inflammation resolved.^97^ Bacterial toxins released during infection induced the loss and disorganization of lymphatic muscle cells in draining vessels, a condition that persisted for at least 120 days post‐infection.^97^

Studies by Kastenmuller et al.^98^ and Lammermann et al.^5^ demonstrated that skin infection with *Pseudomonas aeruginosa* similarly induces a rapid and robust innate immune response in the draining lymph nodes, limiting systemic pathogen spread. Lymph node‐resident macrophages were found to activate natural killer cells, NKT cells, innate‐like CD8^+^ T cells, and γδ T cells by IL‐18 release, leading to rapid IFN‐γ secretion.^98^ Moreover, neutrophil swarming behavior in the lymph node was also observed after skin infection with *Pseudomonas aeruginosa* and this was found to be largely dependent on leukotriene B4.^5^

The skin is certainly one of the easier organs to image, as no surgery is required to image the ear and the dorsal skin flap model only requires minimal invasiveness without opening up any body cavities. Despite having these advantages over imaging other internal organs, there are many unanswered questions that can be addressed using skin IVM. Neutrophils have been studied extensively with IVM, as they are robustly recruited to sites of infection and often play an important role in pathogen clearance. However, neutrophils are not the only immune cell present at sites of infection. Monocytes/macrophages are also recruited at later time points, yet we know very little about where they localize and what their function is. Furthermore, the roles of other innate cells such as iNKT cells or innate lymphoid cells have not been investigated during bacterial skin infections. Skin infections may or may not heal, and understanding the roles that immune cells play in the healing process will be imperative to finding new treatments. One advantage of using multiphoton IVM is the ability to visualize the dense collagen network in the dermis with second harmonic generation.^5^ By incorporating this into IVM studies, the contribution of immune cells and bacterial virulence factors toward the destruction and reformation of collagen throughout infection is possible, providing further insight into the wound healing process.

Moreover, we need a better understanding of how immune cells interact with other cells within the skin, including epithelial cells and stem cells, as this will provide us with a more complete picture of skin physiology and function versus pathology and dysfunction during homeostasis and disease. The skin microbiota plays an important role in maintaining homeostasis. However, recent work has suggested that the microbiota can also augment *Staphylococcus aureus* pathogenesis,^99^ so future imaging studies using germ free or gnotobiotic mice will be important. Burn patients also have a high risk of developing severe infections, including sepsis, due to compromised barrier function and immune dysfunction.^100^ In these patients, both commensal and nosocomially transmitted microorganisms can cause difficult‐to‐treat infections.^100^ Thus, using models of skin injury in combination with infection is needed to understand differences in the pathogenicity of bacteria as well as differences in the innate immune response.

### 3.4 Localized infections: Imaging the urinary tract

Urinary tract infections (UTIs) are one of the most common bacterial infections and continue to be a major medical concern in both the developing and developed world.^101^ The main causative agent is uropathogenic *Escherichia coli* (UPEC), which accounts for more than 65% of infections.^101^ UPEC can persist intracellularly in the urinary tract, causing relapsing infections, and if left untreated can ascend to the kidneys.^102^ The innate immune response to bacteria in the urinary tract during the different phases of infection is becoming better understood. Upon infection, resident Ly6C^−^ macrophages act as sentinels to attract circulating neutrophils and Ly6C^+^ monocytes/macrophages into the uroepithelium.^103^ Crosstalk between resident and recruited macrophages via TNF release has been shown to be required to enhance the initial recruitment of neutrophils to the site of infection.^103^ On the other hand, uptake of bacteria by resident macrophages may actually impede the development of an adaptive immune response during UTI, as the depletion of these cells was shown to enhance bacterial uptake by dendritic cells and reduce bacterial burden upon secondary challenge.^104^

IVM has been utilized to investigate immunologic responses in the urinary tract^105^, ^106^, ^107^, ^108^, ^109^, ^110^, ^111^, ^112^; however, studies focused on infection are more limited.^113^, ^114^, ^115^, ^116^ IVM has helped elucidate the mechanisms that underlie leukocyte recruitment dynamics to the bladder^113^ and pathophysiology in the kidneys^114^, ^115^, ^116^ during infection with *Escherichia coli*. For instance, it was observed that a localized UPEC infection rapidly, yet indirectly, caused microvascular dysfunction and clotting in the kidney, which helped to contain the bacteria within the tubules and prevent dissemination, suggesting a protective mechanism by the host.^115^

There are still many interesting questions that remain to be answered regarding host‐pathogen interactions in the organs of the urinary system. The lower urinary tract is constantly exposed to microbes from the external environment, yet generally resists infection.^117^ This is largely dependent on innate immune responses as adaptive immune responses in the urinary tract are limited and, thus, recurring infections in humans are common.^101^, ^117^ IVM could be used to better understand the complex relationship between various resident and recruited immune cells in balancing the beneficial clearance of bacteria versus developing effective adaptive immunity at this site. Additionally, the bladder has recently been found to have its own microbiome, contrary to previous dogma that it is sterile.^118^, ^119^, ^120^ Thus, future work to understand host‐microbiome interactions in the urinary tract during health and infection are needed.

Bacteria are able to move from the bladder to the kidney during the natural course of infection and a commonly used model of upper UTI is the direct delivery of bacteria into the tubules of the kidney by micropuncture.^114^, ^115^, ^116^ Although a feasible and reproducible model, it does not reflect the natural transition from one niche to another, which has been shown to influence the pathogenicity of UPEC.^121^ Thus, future IVM studies, perhaps using multiple chronic windows, to visualize immune responses over the course of ascending UTI would be very interesting. Moreover, a clear sex bias in the risk and prevalence of UTIs exists, with women being much more susceptible than men.^122^ Although this disparity is largely ascribed to anatomic differences, mounting evidence suggests that estrogen and other sex‐specific molecules broadly influence the host immune response.^122^ Hence, future studies to tease apart the influence of sex hormones on host‐pathogen responses in the urinary tract are warranted.

## 4 OTHER ORGAN SYSTEMS

IVM has been used to study many different organ and animal systems. Murine models are typically used for IVM studies; however, this technique has also been utilized to study tissues in animals such as rats, zebrafish, *Caenoerhabditis elegans*, and *Drosophila melanogaster*.^3^ In this review, we specifically discussed imaging of the lungs, spleen, liver, brain, joints, GI tract, skin and skin‐draining lymph nodes, and urinary tract to better understand host‐pathogen interactions during infection. However, other tissues are also amenable to IVM, including the bone marrow, eyes, adipose tissue, spinal cord, placenta, and cremaster muscle. The cremaster muscle has been widely imaged to visualize leukocyte‐endothelial cell interactions and leukocyte recruitment.^123^ Although this tissue has provided invaluable information on fundamental immunology, including immune responses to infection, it is clear that not all findings can be generalized to other organs, perhaps in part due to local environments affecting the phenotype of tissue‐specific endothelium. Technologic advances of IVM have allowed researchers to ask tissue‐specific questions regarding host‐pathogen responses in most organs and a common conclusion is that each organ has its own signature. Moreover, it is becoming more common to perform system‐wide analyses and studies are utilizing the powerful ability of IVM to visualize real‐time cellular dynamics in order to screen responses in multiple organs. The zebrafish embryo is particularly amenable to in vivo IVM, as it can be imaged in its entirety to visualize immune cells throughout the body of this more primitive translucent organism.^124^ Because “seeing is believing,” there is little doubt that IVM will continue to provide valuable insight into host‐pathogen interactions in a range of different tissues and organisms over the course of infection with different types of microbes.

## 5 CONCLUDING REMARKS

Although IVM has opened our eyes to many different types of cell‐cell and cell‐microbe interactions, we can only see what we label. We are still limited in our ability to specifically label various immune and nonhematopoietic cells simultaneously for imaging and therefore typically need to confirm our results with other techniques such as flow cytometry. Moreover, the loss of fluorescence signals due to photobleaching or cell division has also been a tremendous roadblock in the imaging field. However, as new markers are validated, more strains of transgenic mice are generated, and microscopes are improved, these issues may soon be problems of the past. Notably, the advancement of label‐free imaging technologies offers exciting new possibilities for the field of IVM. Second, and now third, harmonic generation, for example, allows the visualization of structures like collagen and blood flow without dyes.^125^

One of the biggest limitations of IVM is that, in most cases, only one time point in the course of an infection can be imaged in a single animal. With the advent of the chronic window, new insights into the spatiotemporal regulation of the immune system at multiple time points in the same animal can be established. The implantation of a coverslip in a metal frame provides long‐term optical access to a tissue of interest.^126^ For example, the chronic cranial window is permanently secured to the skull following a craniotomy to expose an area of the brain, allowing researchers to image the same location for up to a year.^126^ To date, chronic windows have been developed for the brain, skin, lymph node, liver, spleen, and other abdominal organs, and most recently, the lung.^126^, ^127^ Although chronic windows can induce some inflammation when the window is implanted, using this technique to study chronic infections, such as tuberculosis or those seen in cystic fibrosis patients who are plagued by long‐term staphylococcal and *Pseudomonas* infections, would be of great interest to the medical community. Ideally one would like to be able to image humans to see the immune system at play in our own bodies. Although the field continues to move in that direction, for example, with humanized mice (mice with functional human cells and tissues), imaging the immune system in humans directly remains the panacea.

An exciting area in the realm of host‐microbe interactions is the study of germ‐free and gnotobiotic mice. It is becoming a priority in some germ‐free facilities to install intravital microscopes in the sterile environment to ensure that the animals remain naïve to outside bacteria during imaging. These types of studies will allow scientists to answer very interesting questions about how the immune system develops, surveys, and responds when the microbiota is absent or altered. Although this review focused on bacterial infections, IVM of viral and fungal infections, and co‐infections across kingdoms, are becoming more common.

While academic innovations pave the way for future applications of IVM, support from the biotech and bioinformatic industries could mean access to better tools for researchers. As the ability to image more cell types over longer periods of time improves, the amount of data collected will exponentially increase, changing the way investigators will need to approach analyses. Moreover, combining IVM with mathematical modeling may also be a useful approach to help answer questions of greater complexity in the fields of infectious disease and immunity.

IVM has been an invaluable tool for scientists in many fields ranging from immunology, infectious disease, neuroscience, to cancer. It has supported numerous seminal discoveries in immunology and improved our understanding of host‐microbe interactions; however, there are still many questions that need answering. For example, how do bacteria move from a localized infection site to become systemic? When neutrophils are recruited to an infection site, do they all come from the blood and bone marrow, or do they leave other reservoirs such as the spleen and lungs? How is inflammation resolved after infections are cleared? Do all immune cells die at the site of infection or do they disseminate pathogens to other organs? Looking back, IVM has certainly come a long way from the early 1800s and the next decade is sure to bring increasingly important, elegant, and interesting findings.
